# Deterioration after corticosteroids in CIDP may be associated with pure focal demyelination pattern

**DOI:** 10.1186/1471-2377-14-72

**Published:** 2014-04-04

**Authors:** Filip Eftimov, Marinus H Liesdek, Camillus Verhamme, Ivo N van Schaik

**Affiliations:** 1 Department of Neurology, Academic Medical Center, University of Amsterdam, Amsterdam, DD, 1100, The Netherlands

**Keywords:** Chronic inflammatory demyelinating polyneuropathy, CIDP, Deterioration, Corticosteroids

## Abstract

**Background:**

In the PREDICT study, a randomised controlled trial comparing dexamethasone with prednisolone in patients with chronic inflammatory demyelinating polyradiculoneuropathy (CIDP), almost a quarter of patients deteriorated soon after starting treatment. The primary objective of this post-hoc analysis was to test the hypothesis that a focal demyelination pattern is associated with early deterioration after corticosteroid treatment and to explore whether various clinical characteristics are associated with deterioration after corticosteroid treatment.

**Methods:**

Clinical outcome was categorised into early deterioration and non-early deterioration. A neurophysiologist blinded for treatment outcome scored electrophysiological data into following categories: pure focal versus non-focal distribution of demyelination and no/minor versus moderate/severe sensory involvement. Additionally, we compared electrophysiological and clinical baseline parameters, with emphasis on previously reported possible associations.

**Results:**

Early deterioration was found in 7 out of 33 patients (21%). Ten patients had pure focal distribution of demyelination, of whom 5 had early deterioration; 23 patients had non-focal distribution, of whom 2 had early deterioration (p = 0.02). Higher mean median nerve sensory nerve conduction velocity (SNCV) was found in patients with early deterioration compared to patients with non-early deterioration (52.6 and respectively 40.8 m/s, p = 0.02).

**Conclusion:**

Pure focal distribution of demyelination and lesser sensory electrophysiological abnormalities may be associated with early deterioration in CIDP patients treated with corticosteroids.

## Background

Corticosteroids and intravenous immunoglobulin (IVIg) are both efficacious treatments for chronic inflammatory demyelinating polyradiculoneuropathy (CIDP). However, first choice of treatment is often based on physicians’ preference, due to absence of difference in treatment efficacy [1,2]. Predicting factors of treatment response in CIDP would greatly ease the choice of first treatment. Thus far, reliable clinical and electrophysiological predictors of treatment response are limited.

Earlier, we performed the PREDICT trial in which remission rates were compared between pulsed dexamethasone treatment and daily prednisolone in typical CIDP patients with diffuse sensory and motor involvement [3]. Forty percent of the whole group achieved a remission, defined as a sustained improvement one year after start of treatment, without a significant difference between both treatments. Strikingly, almost a quarter of the participants deteriorated soon after start of trial treatment.

Early deterioration after corticosteroids is a well-recognised and enigmatic phenomenon reported in patients with multifocal motor neuropathy (MMN) [4]. To a lesser extent, deterioration after corticosteroids has also been reported in pure motor CIDP and Lewis-Sumner syndrome (LSS) [5,6]. The hallmark of MMN is the presence of conduction blocks (CBs) caused by focal demyelination, while signs of diffuse demyelination such as reduced motor nerve conduction velocities (MNCVs) are less frequently found [7]. This is in contrast with typical motor and sensory CIDP in which both diffuse and focal features can occur. Some studies have suggested that in pure motor CIDP and LSS conduction blocks are relatively more frequent than other demyelinating features, when compared to typical CIDP [6,8]. These findings prompted us to consider an association between deterioration after corticosteroids and the presence of CBs with focal demyelination pattern in acquired demyelinating neuropathies, including typical CIDP.

A few small retrospective studies have reported an association between extent of axonal damage or extent of sensory involvement and treatment response after corticosteroids, but none have focussed on early deterioration [9-11]. In this post-hoc analysis we studied possible associations with deterioration after corticosteroid treatment, focussing on the pattern of demyelination and previously reported clinical and electrophysiological features associated with poor response to corticosteroid treatment [10,11].

## Methods

### Patients and treatment

The PREDICT trial included treatment naïve patients who had been diagnosed as having definite or probable CIDP according to the ENMC diagnostic criteria [12]. All patients had a typical CIDP with clinically symmetrical sensory and motor involvement. Patients with a pure motor CIDP or LSS were excluded from the trial. Participants were randomly assigned to receive either six monthly courses of oral pulsed dexamethasone (40 mg daily during four consecutive days) or 8 months daily prednisolone with a tapering schedule [3]. All participants gave written informed consent for the original trial including analysis of baseline characteristics. The trial protocol was approved by the ethics committees of all participating centres.

### Treatment outcome

In the PREDICT trial remission was the primary outcome defined as improvement of at least three points on the Rivermead Mobility Index (RMI) and improvement of at least one point on the Inflammatory Neuropathy Cause and Treatment (INCAT) disability scale as compared with baseline or when the best possible score of a scale had been reached. For this post-hoc analysis we dichotomised outcome into early deterioration and non-early deterioration. Early deterioration was defined as any decrease on the RMI and/or increase on the INCAT disability scale at first scheduled trial follow-up visit at 8 weeks or at a prior unscheduled visit if such occurred. The group of non-early deterioration group encompassed all other patients.

### Baseline characteristics

Electrophysiological baseline parameters were re-assessed and scored into categories by a neurophysiologist blinded for treatment and treatment outcome. The EFNS/PNS criteria for CIDP were used to define a conduction block and number of segments with a MNCV within the demyelinating range (<70% of lower limit of normal) [2]. Nerve segments over pressure points (elbow and fibula) and nerves with distal compound muscle action potential (CMAP) peak-peak amplitude below 1 mV were excluded from analysis [2]. The number of CBs was defined as the sum of definite and probable CBs and was expressed as number per patient and as number per measured nerve segments with distal CMAP amplitude above 1 mV. The mean MNCV in arms and legs was calculated using exclusively nerve segments without CB. The distal CMAP amplitude and sensory nerve action potential (SNAP) amplitude of the median nerve were expressed as means. If both median nerves were tested in a patient, the mean of these values was used for group comparison. In non-recordable median nerves the CMAP and SNAP was scored as 0.

Categorization of pure focal versus non-focal pattern of demyelination was based on the assumption that CB is a sign of focal demyelination while reduced MNCV in a nerve segment without CB is a sign of diffuse demyelination. We defined a pure focal demyelination pattern as the presence of two or more definite or probable CBs without the presence of MNCVs within the demyelinating range in other nerve segments. All other patients were considered having a non-focal demyelinating pattern.

Electrophysiological sensory involvement at baseline was categorised into no/minor versus moderate/severe. Moderate/severe sensory involvement was defined as the presence of SNAP amplitude below the lower limits of normal (LLN) in at least two arm nerves. If a single arm nerve was tested, moderate/severe sensory involvement was scored if this single SNAP amplitude was below the LNN. All other patients were scored as having minor sensory involvement. Sensory nerve conduction velocity (SNCV) of the median nerve was calculated if distal and proximal SNAP recordings were available.

Clinical baseline characteristics included Medical Research Council (MRC) sum score of 12 predefined muscle groups (maximum of 60), grip strength expressed as mean grip strength from both hands measured with a Vigorimeter, INCAT sensory sum score (range 0-20), RMI (range 0-15) and INCAT disability (range 0-10) scores at baseline.

### Statistical analysis

Differences between baseline characteristics were tested for significance using chi-square test and, if needed, a Fisher exact test for nominal data, Student’s t-test for interval data with normal distribution and Mann-Whitney test for all other data (expressed as median values), using a p value of .05 as threshold for statistical significance.

## Results

Forty patients participated in the PREDICT trial. Seven patients had an alternative diagnosis during follow-up and were excluded from analysis. Alternative diagnoses included hereditary neuropathy, plasmacytoma, testicular lymphoma, IgM paraproteinemia and transthyretine associated hereditary amyloidosis. Details on these patients have been published elsewhere [13].

**Figure 1:**
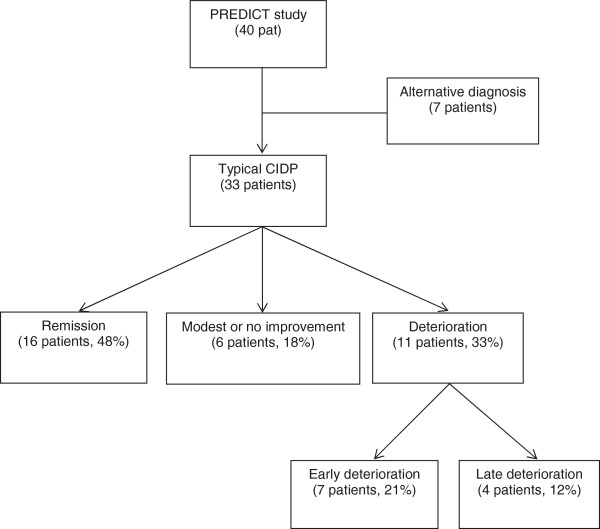
Outcome of patients during PREDICT study.

From 33 CIDP patients seven (21%) deteriorated within 8 weeks after start of treatment (Figure 1), four patients had dexamethasone and three had prednisolone. In three of these patients deterioration occurred within two weeks. Clinical characteristics of patients with early deterioration can be found in Table 1. One patient (Table 1, patient 7) had an asymmetric distribution of weakness based on MRC scores and grip strength. All other 32 patients had symmetrical distribution of weakness. In all included patients weakness was present in both arms and legs. Legs were predominantly affected in six patients (all without deterioration), arms were predominantly affected in two (one deteriorated, Table 1, patient 2).

**Table 1:** Clinical characteristics of patients with early deterioration

	**Baseline**			**Time to endpoint (weeks)**	**Change at endpoint compared to baseline**					**Response to other treatments**
	**MRC**	**GS**	**INCAT-SS**		**ΔINCAT-DS**	**ΔRMI**	**ΔMRC**	**ΔGS**	**Δ INCAT-SS**	
1/D	48	51	np	5	1	-4	-2	-15	np^a^	P
2/D	53	108	5	6	1	0	5	-52	-3	No
3/D	47	18	3	1	3	-7	-5	-5	1	IVIg
4/D	52	125	2	1	4	-10	-7	-25	0	IVIg
5/P	48	47	7	8	4	-7	-6	-27	-2	IVIg
6/P	50	9	11	2	3	-2	np	np	np	P
7/P	51	83	9	4	2	0	1	-47	5	No

Legend: *Abbreviations*: *D* dexamethasone, *P* prednisolone, np not performed, *MRC* Medical Research Council Sum Score (maximum of 60), *MGS* Mean Grip Strength (mean of both arms, Vigorimeter) *INCAT-DS*, Inflammatory Neuropathy Cause and Treatment Disability Scale (maximum score of 10, higher scores represent more disability), *RMI* Rivermead Mobility Index (maximum score of 15, higher scores represent less disability), *INCAT-SS* Inflammatory Neuropathy Cause and Treatment Sensory Sum Score (maximum score of 20, higher scores represent more sensory deficit). ^a^INCAT-SS in this patient at time of deterioration was 10.

From the seven patients who deteriorated, five patients had ultimately a favorable outcome (Table 1). Three patients responded to IVIg, one patient failed to improve on IVIg but improved after switch to prednisolone, one patient (prednisolone) had only a temporary deterioration, continued prednisolone treatment which later resulted in a remission. Two patients did not respond to any treatment, including various immunosuppressive agents. Of the 26 remaining patients, four patients (all prednisolone) deteriorated during or after tapering of prednisolone (10 to 33 weeks after start of treatment, Figure 1).

All patients fulfilled the electrophysiological EFNS/PNS criteria for CIDP [2]. The median number of examined motor nerve segments per patient was 7 (IQR 4-12, with a median of 6 segments with a CMAP peak-peak amplitude above 1 mV). Pure focal distribution pattern of demyelination was found in 10 patients. Of these patients, CB was the only demyelinating feature in seven patients while three patients had also abnormal F-responses in nerves without a conduction block and/or prolonged distal latencies.

The median number of examined sensory arm nerves per patient was 3 (IQR 2-4). In three patients only one sensory arm was tested. Two patients had normal sensory conduction studies, based on a limited number of tested nerves. Both patients had clinically sensory involvement with INCAT sensory sum scores of 2 and 11 and both deteriorated (patient 4 and 6 in Table 1). All other patients had electrophysiological sensory abnormalities.

Five out of seven (71%) patients with early deterioration had a focal distribution pattern of demyelination, compared to five out of 26 (19%) patients without early deterioration (p = 0.02, Table 2). Total number of conduction blocks per examined nerve segment, median CMAP amplitude of the median nerve and mean MNCV did not differ between both groups.

**Table 2:** Distribution of baseline characteristics according to treatment outcome

	**Early deterioration (7 patients)**	**Non-early deterioration (26 patients)**	**p value**
**Baseline electrophysiological parameters**			
Pattern of demyelination, number (%)			
Pure focal	5 (71%)	5 (19%)	0.02
Non-Focal	2 (29%)	21 (81%)	
CMAP amplitude median nerve (median, mV)	10 (6)	6 (5)	0.29
MNCV arms (mean, m/s)	49.7 (10)	39.6 (12)	0.08
MNCV legs (mean, m/s)	39.6 (3)	37.1 (12)	0.53
Number of CB per examined segments (mean)	0.31 (0.2)	0.36 (0.2)	0.70
Sensory involvement severity, number (%)			
Normal/minor	4 (57%)	6 (23%)	0.19
Moderate/severe	3 (43%)	20 (77%)	
SNAP amplitude median nerve (median, μV)	15 (13)	7 (10)	0.13
SNCV median nerve (mean m/s)	52.6 (7)	40.8 (12)	0.02
**Baseline clinical parameters**			
MRC sum scores (mean)	49.9(2)	50.2 (5)	0.77
Grip strength (median, kPa)	102 (180)	88 (64)	0.37
INCAT sensory sum score (median)	6(7)	9(7)	0.56
INCAT disability score (median)	3(2)	4(3)	0.42
Rivermead Mobility Index (median)	12(3)	12(6)	0.91

Legend: *Abbreviations*: *MNCV* motor nerve conduction velocity, *CB* conduction block (definite and probable summated), *MRC* Medical Research Council, *INCAT* inflammatory neuropathy cause and treatment. Numbers in brackets represent the standard deviation for mean values or interquartile range for median values.

Mean SNCV in median nerve was higher in patients with early deterioration compared to other patients (52.6 and 40.8 m/s respectively, p = 0.02). There was no difference in the predefined categories of sensory involvement and the median SNAP amplitude of the median nerve between both groups.

## Discussion

In this post-hoc analysis we found that pure focal demyelination and higher mean SNCV of the median nerve were associated with early clinical deterioration in patients with CIDP after starting corticosteroid treatment. No association was found with other electrophysiologal and clinical baseline parameters.

Deterioration in patients with CIDP might be due to progression of disease and/or insufficient therapeutic effect of corticosteroids. Most patients in the PREDICT trial who deteriorated however, worsened dramatically within weeks after start of treatment. Furthermore, all PREDICT-patients were treated with pulses of dexamethasone or were receiving high doses of daily prednisolone at the time of deterioration. In a recent trial comparing IVIg with intravenous methylprednisolone in CIDP a comparable percentage of patients (24%) deteriorated after start of pulses intravenous methylprednisolone [14].

Earlier studies have suggested that higher MNCV, higher SNCV and higher SNAP amplitudes are associated with poor corticosteroid response [9-11]. In our study we focussed on early deterioration instead of poor response but our results are in line with previous findings as we also found higher mean SNCV in patients who deteriorated. These findings suggest that an electrophysiological profile with predominantly motor involvement with focal demyelination might be a risk factor for deterioration after corticosteroid treatment. Moreover, our data further suggests that it might be difficult to identify patients likely to deteriorate based on clinical characteristics as all patients had a typical clinical picture of CIDP with diffuse symmetric sensory and motor involvement, although discrepancy between clinical findings of evident motor involvement and only minor sensory involvement was found in two patients who deteriorated (Table 1).

Except for myasthenia gravis we are not aware of any other immune-mediated diseases in which corticosteroids are known to have such a detrimental effect. This prompted us to consider corticosteroid-induced changes in nerve conduction rather than disease progression due to lack of immunological response to corticosteroids. Threshold techniques have shown that conduction in inflammatory neuropathies can fail if membrane potential hyperpolarizes too far from threshold. In general, motor nerves are more vulnerable than sensory nerves that are able to counteract hyperpolarization. Critically conducting motor nerves, as can be seen in nerves with conduction blocks, might be especially vulnerable for changes in rest membrane potential [15,16]. This vulnerability of motor nerves might explain why in most of our patients increase in disability was accompanied by deterioration on MRC sum score and/or grip strength rather than increase of sensory deficit (Table 1). A possible mechanism of this detrimental effect of corticosteroids might be axonal hyperpolarization by up-regulation of Na^+^K^+^ pump activity. Up-regulation of hyperpolarizing Na^+^K^+^ pumps have been recently shown in muscle fibres from muscle biopsies from healthy volunteers treated with a course of dexamethasone [17]. Studies on effect of corticosteroids on nerve conduction and membrane potentials have not been published.

Unfortunately, the number of patients in this study is too small to provide solid evidence on associations between baseline characteristics and treatment outcome. The main limitation of the study is possible bias associated with a post-hoc analysis. This bias was somewhat limited as patients were assessed during prescheduled follow-up visits and predefined measurements and treatment outcome. Another important limitation of this study is the lack of standardised electroneurography protocols which led to testing of different nerves segments and variable numbers of examined nerve segments. Furthermore, most electroneurography protocols focussed on examination of motor nerves as abnormalities in these nerves are needed to meet the electrophysiological criteria for CIDP. This has resulted in limited number of examined sensory nerves in some participants. To overcome some of these limitations we made categories of distribution of demyelination and sensory involvement which were assessed by a neurophysiologist blinded for treatment outcome. Considering the explorative nature of this study, categories were based on the presence of conduction blocks indicating focal demyelination and low MNCV indicating diffuse demyelination in a nerve segment. We chose not to use abnormal or absent F-waves or prolonged distal motor nerve latencies for this category distinction as one could argue whether these are focal or non-focal features. To explore possible bias we also performed an analysis in which focal demyelination was defined as CB without any other demyelinating features. This analysis led to similar results (data not shown).

Finally, we performed analyses including all PREDICT participants and deterioration throughout the whole trial period to explore possible bias due to exclusion of patients with alternative diagnosis or our definition of cut-off time point for early deterioration. Significant association with deterioration was still present with focal demyelination pattern, but not for SNCV.

Larger studies are needed to confirm the association of focal pattern of demyelination and early deterioration. Extensive electroneurography protocols might be useful, not only to confirm diagnosis, but also to explore the predictive value of electrophysiological phenotypes for treatment response. Identifying patients who deteriorate after treatment is important as IVIg can be administered as alternative treatment. We did not find a difference in number of CBs between both groups but CBs might be more frequent in responders to IVIg compared to non-responders [18]. When combining these results, in patients with clinically typical CIDP and a pure focal demyelination pattern IVIg might be the preferred treatment rather than corticosteroids. Alternatively, in CIDP patients with a non-focal pattern of demyelination, corticosteroids can safely be administered, which has advances over IVIg as there is increasing evidence that long-term remission can be achieved after a relatively short period of treatment with corticosteroids [13,14].

## Conclusions

Focal pattern of demyelination and lesser sensory electrophysiological abnormalities may be associated with deterioration after corticosteroid treatment in typical CIDP. Further studies are needed to confirm these associations and to explore the predictive value of different electrophysiological phenotypes for treatment response.

## Abbreviations

CIDP: Chronic inflammatory demyelinating polyradiculoneuropathy; IVIg: Intravenous immunoglobulin; MMN: Multifocal motor neuropathy; LSS: Lewis-sumner syndrome; CB: Conduction block; MNCV: Motor nerve conduction velocity; CMAP: Compound muscle action potential; SNAP: Sensory nerve action potential; SNCV: Sensory nerve conduction velocity; RMI: Rivermead mobility index; INCAT: Inflammatory neuropathy cause and treatment; MRC: Medical research council; LLN: Lower limits of normal.

## Competing interests

M Liesdek and Dr Verhamme report no disclosures. Dr. F. Eftimov received a honorarium for a single interview on immunoglobulin therapy in CIDP. Dr. van Schaik received departmental honoraria for serving on scientific advisory boards and a steering committee for CSL-Behring. He received departmental research support from The Netherlands Organisation for Scientific Research, and from the Dutch Prinses Beatrix Fonds. All lecturing and consulting fees were donated to the Stichting Klinische Neurologie, a local foundation that supports research in the field of neurological disorders.

## Authors’ contribution

FE, CV and IvS were involved in study design, data acquisition, and analysis and interpretation of the data. ML participated in data acquisition and analysis. FE drafted the manuscript which was revised by ML, CV and IvS. All co-investigators from the PREDICT study group participated on data acquisition. All authors read and approved the final manuscript.

## Pre-publication history

The pre-publication history for this paper can be accessed here:

http://www.biomedcentral.com/1471-2377/14/72/prepub
